# Pristine and Modified Porous Membranes for Zinc Slurry–Air Flow Battery

**DOI:** 10.3390/molecules26134062

**Published:** 2021-07-02

**Authors:** Misgina Tilahun Tsehaye, Getachew Teklay Gebreslassie, Nak Heon Choi, Diego Milian, Vincent Martin, Peter Fischer, Jens Tübke, Nadia El Kissi, Mateusz L. Donten, Fannie Alloin, Cristina Iojoiu

**Affiliations:** 1 Univ. Grenoble Alpes, Univ. Savoie Mont Blanc, CNRS, Grenoble INP, LEPMI, 38000 Grenoble, France; misgina-tilahun.tsehaye@grenoble-inp.fr (M.T.T.); getachewtek0@gmail.com (G.T.G.); vincent.martin@lepmi.grenoble-inp.fr (V.M.); 2 Applied Electrochemistry, Fraunhofer Institute for Chemical Technology ICT, Joseph-von-Fraunhofer, Straße 7, 76327 Pfinztal, Germany; nak.choi@ict.fraunhofer.de (N.H.C.); peter.fischer@ict.fraunhofer.de (P.F.); jens.tuebke@ict.fraunhofer.de (J.T.); 3 Institute for Mechanical Process Engineering and Mechanics, Karlsruhe Institute of Technology KIT, Straße am Forum 8, 76131 Karlsruhe, Germany; 4 Univ. Grenoble Alpes, CNRS, Grenoble INP, LRP, 38000 Grenoble, France; diego.milian@univ-grenoble-alpes.fr (D.M.); Nadia.ElKissi@ujf-grenoble.fr (N.E.K.); 5 Amer-Sil S.A., 61 Rue d’Olm, 8281 Kehlen, Luxembourg; mateusz.donten@amer-sil.com; 6 Réseau sur le Stockage Electrochimique de l’Energie (RS2E), CNRS, FR3459, CEDEX, 80039 Amiens, France

**Keywords:** zinc slurry–air flow battery, membrane coating, UV irradiation, zincate crossover, power density

## Abstract

The membrane is a crucial component of Zn slurry–air flow battery since it provides ionic conductivity between the electrodes while avoiding the mixing of the two compartments. Herein, six commercial membranes (Cellophane™ 350PØØ, Zirfon^®^, Fumatech^®^ PBI, Celgard^®^ 3501, 3401 and 5550) were first characterized in terms of electrolyte uptake, ion conductivity and zincate ion crossover, and tested in Zn slurry–air flow battery. The peak power density of the battery employing the membranes was found to depend on the in-situ cell resistance. Among them, the cell using Celgard^®^ 3501 membrane, with in-situ area resistance of 2 Ω cm^2^ at room temperature displayed the highest peak power density (90 mW cm^−2^). However, due to the porous nature of most of these membranes, a significant crossover of zincate ions was observed. To address this issue, an ion-selective ionomer containing modified poly(phenylene oxide) (PPO) and *N*-spirocyclic quaternary ammonium monomer was coated on a Celgard^®^ 3501 membrane and crosslinked via UV irradiation (PPO-3.45 + 3501). Moreover, commercial FAA-3 solutions (FAA, Fumatech) were coated for comparison purpose. The successful impregnation of the membrane with the anion-exchange polymers was confirmed by SEM, FTIR and Hg porosimetry. The PPO-3.45 + 3501 membrane exhibited 18 times lower zincate ions crossover compared to that of the pristine membrane (5.2 × 10^−13^ vs. 9.2 × 10^−12^ m^2^ s^−1^). With low zincate ions crossover and a peak power density of 66 mW cm^−2^, the prepared membrane is a suitable candidate for rechargeable Zn slurry–air flow batteries.

## 1. Introduction

Redox-flow batteries (RFBs) are promising electrochemical energy storage devices for mitigating the intermittent fluctuation of solar and wind power plants [1,2]. These batteries offer several advantages, such as independent sizing of power and energy, room temperature operation, scalability, long charge/discharge cycle life and high efficiency [3]. Over the years, various types of RFBs, such as vanadium-based RFBs [4,5,6] and metal–air flow batteries have been developed [7,8,9]. Particularly, Zn–air battery presents a high potential for mobile and stationary applications because of its high theoretical energy density (1087 Wh kg^−1^, oxygen inclusive), abundant raw materials, environmental friendliness and economic viability [10,11,12,13]. To improve its cycling and discharge performance, various studies have mainly focused on preparation and improvement of Zn electrode [14,15,16], electrocatalyst air electrodes [17,18] and electrolyte formulations [7,19].

In such batteries, the Zn electrode can be a semi-solid, fluidic electrode, in which particles are mixed into the electrolyte to form a slurry [9]. In other words, the Zn slurry (Zn particles suspended in alkaline electrolytes) is used as both the anode electrode and electrolyte [20,21]. In these batteries, unlike conventional Zn–air batteries, the volume of tank or the amount/concentration of Zn particles in the slurry, rather than the size of the porous Zn electrode used in the system, determine the capacity of the battery [22,23]. Moreover, such Zn slurry-based configuration is believed to minimize formation of dendrites and surface passivation since the negative electrode acts only as a current collector [9,23,24,25], thus enhance battery performance.

However, some issues, such as full utilization of the Zn particles in the electrochemical reaction, blockage of the Zn particles in the electrode [9], integral battery configuration [26] and appropriate membrane development [27,28] have been impeding the development and commercialization of rechargeable Zn slurry–air flow batteries.

The membrane is used for OH^−^ ion conduction and avoiding mixing of the positive and negative active materials. To achieve this, membrane with high alkaline stability, OH^−^ conductivity, mechanical stability and low/no crossover of zincate (Zn(OH)_4_^2−^) ions is required. The overall performance and economic viability of this battery are greatly affected by the properties of the membrane employed [27]. Commercial Zn–air batteries usually use porous polyolefin-based membranes. An excellent review on porous membranes for batteries has been published [29]. One of the disadvantages of these types of porous membranes in RFBs is the crossover active species [30]. Thus, in Zn–air batteries, the soluble Zn(OH)_4_^2−^ ion can pass through the membranes to the air electrode, where the Zn(OH)_4_^2−^ can be converted to ZnO, depending on the pH, (Zn(OH)_4_^2−^ → ZnO (s) + H_2_O + 2OH^−^). The formation of ZnO layers has been reported to cause loss of battery capacity [31] and large cell polarization [11] (as the ZnO powers clog the porous air electrode). Therefore, there is a need for minimizing the crossover of Zn(OH)_4_^2−^ ions through the membranes by optimizing their porosity, pore size and pore size distribution.

To address this issue, the use of anion exchange membranes (AEMs) [27,31,32,33], inorganic-filling [34] or polymer-coating of porous membranes [35,36] have been proposed. For the former, the development of alkaline stable AEMs with well-defined and controlled ionic channel size to improve its selectivity without reducing the ionic conductivity is required. Abbasi et al. [31] prepared benzylic quaternized AEMs using poly (2,6-dimethyl-1,4-phenylene oxide) (PPO) and trimethylamine (TMA) and investigated its Zn–air battery discharge performance (specific discharge capacity of ~800 mAh g^−1^_Zn_). The PPO–TMA membrane exhibited a low Zn(OH)_4_^2−^ diffusion coefficient of 1.1 × 10^−8^ cm^2^ min^−1^.

Another promising strategy, which is rarely used in Zn–air batteries, is surface modification of porous membranes. One way to achieve this is to coat a thin ion-selective polymer layer. The coat is expected to allow OH^−^ transfer through the membrane and minimizes the migration of Zn(OH)_4_^2−^ ions to the cathode compartment without significantly affecting the ion conductivity. Coating of Celgard^®^ membranes with Nafion^®^ 117 solution [35] and polymerized ionic liquid [36] have been reported in the literature. Other than these two studies, the method remains to be not explored and not tested in membranes for Zn slurry–air flow batteries. Moreover, mostly polypropylene (PP)-based Celgard^®^ membranes have been explored, while other commercial porous membranes performance in such batteries remain to be not well studied.

The objectives of the present work were to (i) investigate the performance of several commercial membranes in Zn slurry–air flow battery and screen out appropriate membrane for the application, and (ii) coat the porous membranes with anion-exchange polymers to improve their selectivity. First, six commercial membranes were ex situ characterized in terms of electrolyte uptake, ion conductivity and Zn(OH)_4_^2−^ ions crossover and then tested in a 25 cm^2^ Zn slurry–air flow battery. Aiming at decreasing the crossover of Zn(OH)_4_^2−^ ions, Celgard^®^ 3501 membrane was modified with two different anion exchange polymers. A solution of quaternized PPO and *N*,*N*-diallylpiperidinium chloride (DAPCl) was cast on the top surface of the porous membrane and cross-linked via UV irradiation in the presence of a photo-initiator. Moreover, a commercial anion exchange ionomer, Fumion FAA-3-SOLUT-10 (Fumatech, Germany) was used to modify the same support membrane for comparison purpose. DAPCl was chosen because of its high alkaline stability [37,38]. Similar UV irradiation technique for coating *N*-spirocyclic quaternary ammonium monomer-based ionomer on Tetratex^®^PTFE porous substrate has been reported recently elsewhere [39].

## 2. Results and Discussion

### 2.1. Characterization of Commercial Membranes

#### 2.1.1. Electrolyte Uptake and Ion Conductivity

Six commercial membranes (Celgard^®^ 3501, Celgard^®^ 3401, Celgard^®^ 5550, Cellophane™ 350 PØØ, PBI^®^ and Zirfon^®^) were characterized in terms of electrolyte uptake and zincate ions crossover and were tested in Zn slurry–air flow battery. The composition, nature and structure of these commercial membranes are summarized in Table 1. The pore sizes of the different membranes (Table 1) are sub-micrometric, thus the zinc particles are about three orders of magnitude larger, indicating that there is no risk of metallic zinc crossover in the RFB.

Liquid electrolyte uptake: The wettability of the membranes with electrolyte affect both electrolyte filling time and the ability to retain the electrolyte solution, thus affecting the overall performance of the battery [43,44]. The wettability of a membrane, usually investigated by contact angle measurement, depends on various parameters, such as the chemical affinity between the membrane surface and the electrolyte, porosity (in the case of porous membranes), surface roughness and viscosity of the liquid electrolyte [45].

In this work, we measured the electrolyte uptake of the membranes (Table 2) and calculated the percentage of porosity filled with the electrolyte by considering the density of 6 M KOH (1.26 g cm^−3^) and the density of the polymers (for example, 0.92 g cm^−3^ for PP-based Celgard membranes) and added these values in Table 2. To determine if the membranes change their volume, which indicate the swelling of the polymer matrix and may affect percentage and size of the pores, the dimensional change of the membranes in 6 M KOH was also investigated. In all studied membranes, no significant volume change was observed (all membranes recorded less than 5% volume change), therefore, it can be considered that the electrolyte uptake was mainly inside the pore structure and the initial % of porosity was used in the calculation of pore filling. Similarly, electrolyte uptake-induced zero dimensional change has been reported for Celgard^®^ 3501 elsewhere [46].

Among the PP-based Celgard^®^ membranes, Celgard^®^ 5550 exhibited the highest electrolyte uptake, followed by Celgard^®^ 3501 and Celgard^®^ 3401 with 113, 98 and 49 wt. %, respectively. As a result, 82% of the total porosity of Celgard^®^ 5550, 76% of Celgard^®^ 3501 and 63% of Celgard^®^ 3401 were found to be filled with the electrolyte. The Celgard^®^ 5550 and Celgard^®^ 3501 membranes have the same pore size, thus the small difference in their electrolyte uptake could be attributed to the membrane morphology (induced by the manufacturing process) and the wettability of the polymer matrices which depends on the surfactant used to modify the surface of pores and thus the hydrophilicity of Celgard^®^ membranes [48,49]. The Celgard^®^ 3401 membrane’s lower electrolyte uptake can be explained by its lower pore size (43 nm) and probably by a less hydrophilic surface modification. Indeed, all the Celgard^®^ membranes tested in this study have surfactant coated on their surfaces, however, the nature of the surfactant is not known.

Cellophane™ 350 PØØ is made up of cellulosic material with acidic functions (–COOH) on the surface. The polymer hydrophilic character and the presence of ionic functions (–COO^−^, K^+^ after neutralization) explain the highest electrolyte uptake (130 wt. %) among the tested membranes. Zirfon^®^ is a composite membrane made up of polysulfone that is a hydrophobic polymer and hydrophilic filler (zirconia) [50]. The Zirfon^®^ membrane is manufactured by film casting and has a thickness of about 500 ± 50 μm and a porosity of 50 ± 10%, according to the datasheet provided by Agfa [42]. Due to its higher pore size (150 ± 50 nm), compared to the other membranes, and the presence of hydrophilic ZrO_2_ fillers (85 wt. %, 22 m^2^ g^−1^), the electrolyte uptake corresponds to 89% of the porosity which indicates high wettability between the composite membrane and the electrolyte. Whereas, due to its dense character, the PBI membrane displayed low electrolyte uptake (36 wt. %).

Moreover, the ion conductivities of the membranes were determined by EIS. The membrane impregnated with a solution of 6 M KOH was sandwiched between two gold electrodes, the upper one is smaller and has a diameter of 2 mm. Accordingly, Celgard^®^ 5550 membrane showed higher ion conductivity (70 ± 5.5 mS cm^−1^) than Celgard^®^ 3501 (17 ± 0.7) and Celgard^®^ 3401 (14 ± 2 mS cm^−1^) proving that the efficiency of ionic conductivity pathway depends on the electrolyte uptake and size of pores (only the porous part of the membrane is conductive). Whereas, ion conductivity of the Cellophane™ 350 PØØ membrane (56 ± 6 mS cm^−1^) was found to be higher than that of Celgard^®^ membranes, which can be associated to its higher hydrophilicity and electrolyte uptake. Zirfon^®^ exhibited the highest ion conductivity (212 ± 7 mS cm^−1^) among the tested membranes in agreement with its high electrolyte uptake. The PBI membrane (has a heterocyclic benzimidazole ring) with the lowest electrolyte uptake exhibited a conductivity of only 5.1 ± 1 mS cm^−1^. It should be noted that the conduction mechanism in PBI has to be different than in the other studied porous membranes, the (–N=) and (–NH–) of the imidazole interact with KOH and participate in the OH^−·^conductivity [51]. On the other hand, the conductivity of the 6 M KOH electrolyte was about 590 mS cm^−1^ at room temperature. However, it should be noted that the determination of the porous membranes resistance is difficult due to (i) the high conductivity of 6 M KOH electrolyte inducing low resistance values and (ii) the difficulty to control the amount of KOH solution on the surface and inside the membrane which can lead to an overestimation (excess of KOH solution on the border of upper electrodes) or underestimation (porosity not completely filled).

#### 2.1.2. Zincate Ions Crossover

**Figure 1:**
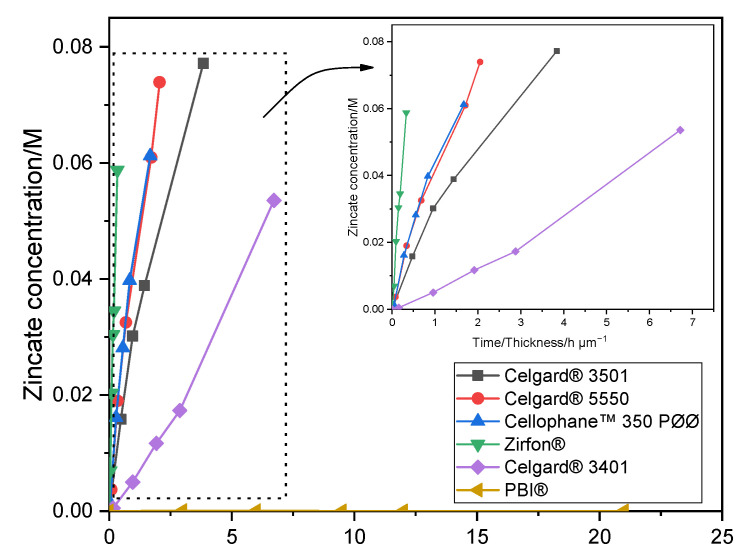
Zn(OH)_4_^2−^ crossover concentration vs. time of Celgard^®^ 3501, Celgard^®^ 3401, Celgard^®^ 5550, Cellophane™ 350 PØØ, and Zirfon^®^ and PBI^®^. Lines in the figure are guides for the eye.

The crossover of soluble Zn(OH)_4_^2−^ ions from the negative to the positive electrodes must be minimized/avoided in order to have working Zn slurry–air battery. Diffusion of Zn(OH)_4_^2−^ through the six membranes was investigated by placing the membrane between the two compartments of the diffusion cells. The amount of Zn(OH)_4_^2−^ ions (M), determined by AAS, crossed through the membranes as a function of testing time/membrane thickness (normalized by membrane thickness) is shown in Figure 1. Celgard^®^ 3501 membrane has the largest diffusion of Zn(OH)_4_^2−^ with a concentration of 0.077 M after 4 days. Considering the 0.3 M of Zn(OH)_2_ used as starting concentration, about 26% of the initial Zn(OH)_4_^2−^ crossed through the membrane in only 4 days, half of the equilibrium concentration, 0.15 M. This can be accredited to the large pore size and porosity, good wettability and small thickness of the membrane. Celgard^®^ 5550 and Cellophane™ 350 PØØ with a concentration of Zn(OH)_4_^2−^ equal to 0.074 and 0.061 M, respectively, exhibited significant crossover within about a week of testing. Whereas, Celgard^®^ 3401 exhibited the lowest Zn(OH)_4_^2−^ ions crossover among the Celgard^®^ membranes. On the other hand, nearly zero crossover of Zn(OH)_4_^2−^ ions was detected in the case of PBI^®^ membrane during the one week of operation, due to its dense and cationic characters. The high crossover of Zn(OH)_4_^2−^ ions through Zirfon^®^ membrane could be due to the high electrolyte channels formed as a result of the large amounts of hydrophilic ZrO_2_ powder within the membranes [40].

To measure and compare the selectiveness of the different membranes, the diffusion coefficient of Zn(OH)_4_^2−^ ions was determined from the slope of the (linear part of the) plot of $\ln\left(\frac{C_{A}}{C_{A}-C_{B}}\right)$ vs. time (Equation (6)) [46]. The results are summarized in Table 3. For comparison purpose, diffusion coefficients of Zn(OH)_4_^2−^ ions of Celgard^®^ 3501 and Cellophane™ 350 PØØ from the literature were included.

As shown in Table 3, the Cellophane™ 350 PØØ, membrane exhibited similar Zn(OH)_4_^2−^ ions diffusion coefficient with that of Celgard^®^ 5550, however, the two membranes have different chemical composition and structure. Cellophane™ membranes have been reported to exhibit lower Zn(OH)_4_^2−^ diffusion coefficient than that of Celgard^®^ 3501 in the literature [53,55,56]. This was suggested to be due to the less porous nature [54] and negative charge [57] of the former membrane than that of Celgard^®^ 3501. However, it must be noted that the measuring protocols (including electrolyte type) and technique might have a significant impact on the diffusion coefficient value as can be seen in the case of Celgard^®^ 3501 in [55] and [52] studies. For instance, Celgard^®^ 3501 and Cellophane™ membranes were reported to have about four times larger Zn(OH)_4_^2−^ ions diffusion coefficient when KOH was used instead of NaOH [56]. Moreover, the Cellophane™ 350POO (Innovia Films Ltd., UK) used in [56] has a thickness of only 25 μm, which is much lower than that of the one used in this study.

In the present study, the Zn(OH)_4_^2−^ diffusion coefficient through the membranes was found to decrease in order as follows: Zirfon^®^ > Celgard^®^ 5550 ≈ Cellophane™ 350 PØØ > Celgard^®^ 3501 > Celgard^®^ 3401 > PBI^®^. This is in agreement with ion conductivities (Section 2.1.1) and porous/dense structure of the membranes.

#### 2.1.3. Zn Slurry–Air Flow Battery Performance

**Figure 2:**
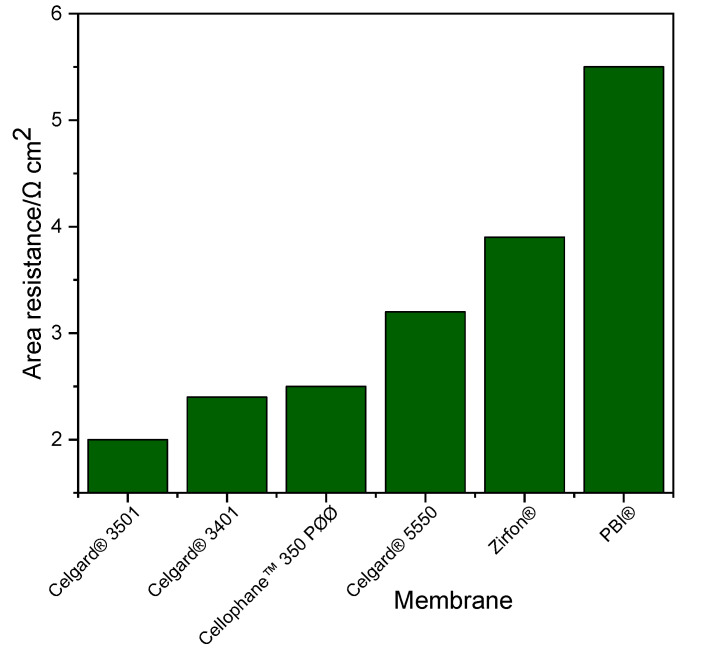
Cell resistance of the Zn slurry–air flow battery employing the different membranes.

Ohmic resistance of the system is a crucial factor in fixing the performance of Zn slurry–air flow battery. Generally, the resistance of the membrane has a large influence on the total resistance of the system. The area resistances of the Zn slurry–air flow battery employing the different membranes was determined from the slope of IV plot and are shown in Figure 2. In addition, similar area resistance results were obtained from the EIS measurements (thus, not included here). The cell with Celgard^®^ 3501 has the lowest area resistance (2 Ω cm^2^) of all the tested cells. Among the Celgard^®^ membranes, despite the high electrolyte uptake, Celgard^®^ 5550 permit to exhibit the highest cell resistance of (3.2 Ω cm^2^) due to its high thickness.

The area resistances obtained in the battery are different from those determined by the conductivity measurement (Section 2.1.1) due to the different electrolytes used. The conductivity of the slurry used in the battery (~261 mS cm^−1^) was about 2.5 times lower than that of the conductivity of the 6 M KOH solution electrolyte (~590 mS cm^−1^) at room temperature. Additionally, the presence of particles in the slurry (Zn and ZnO) can have an impact on the conductivity of the membranes by filling the porosity.

**Figure 3:**
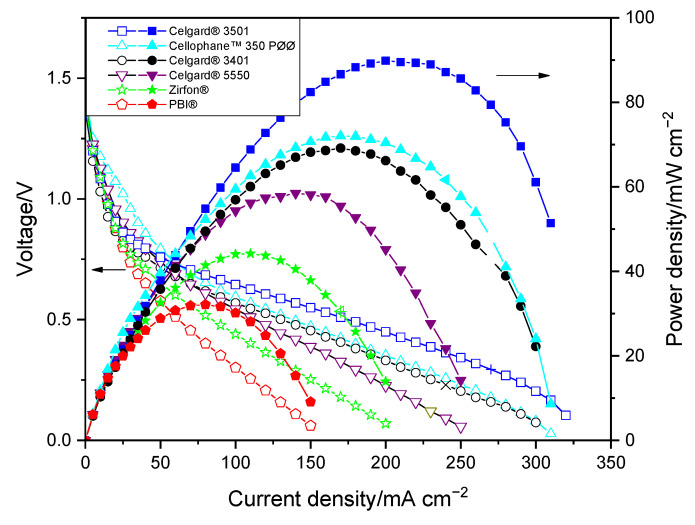
Polarization characteristics of the Zn slurry–air flow battery with the different membranes.

Polarization curves of the membranes in the Zn slurry–air flow battery are shown in Figure 3. The voltage and power density of the cell show significant dependency on the discharge current. A sharp voltage decrease was seen at the beginning of the IV curves for all membranes, which is due to the voltage over potential that is related to the electrochemical reaction on electrodes. The approximately 0.7 V loss at the beginning could be due to the use of an unoptimized air electrode performed without ionomer, which can limit the electrochemical reaction kinetic. In the second part, cell ohmic loss dominates cell performance, leading to a linear decrease in voltage vs. current density [7]. The large decrease in potential at high current density is caused by the concentration polarization (probably due to transport loss into the slurry).

As shown in Figure 3, the limitation associated with mass transport occurred at high current density, thus the maximum power density of the cells seems to be consistent with their in situ area resistances. The discharge cell performance revealed that PBI^®^ and Zirfon^®^-based cells, due to their high area resistances, exhibited the lowest peak power densities among the tested membranes. The PBI^®^-employing cell displayed peak power density of only 32 mW cm^−2^. Among the three Celgard^®^ membranes tested, the cells with Celgard^®^ 3401 and Celgard^®^ 3501 showed maximum peak power density of 69 and 90 mW cm^−2^, respectively, while Celgard^®^ 5550-based cell showed the lowest peak power density (58 mW cm^−2^), consistent with the highest resistance (highest thickness) of the membrane. Cellophane™ 350 PØØ membrane, with an area resistance similar to that of the Celgard^®^ 3401-based cell, displayed a peak power density of 72 mW cm^−2^. It is interesting to note that the peak power densities delivered in decreasing order are Celgard^®^ 3501 > Cellophane™ 350 PØØ ≈ Celgard^®^ 3401 > Celgard^®^ 5550 > Zirfon^®^ > PBI^®^, which is in agreement with the cell resistances obtained.

Overall, Zn(OH)_4_^2−^ ions crossover remains a challenge as it affects the performance and lifespan of Zn slurry–air flow batteries. All the membranes tested, except PBI, present too high crossover of Zn(OH)_4_^2−^ to be used in rechargeable Zn slurry–air flow batteries. Zn(OH)_4_^2−^ crossover leads to capacity loss with cycling and cell polarization as the insoluble ZnO possibly clogs the active area of the positive electrode. However, the critical concentration at which such impact is seen in Zn–air batteries has not been investigated yet. For Zn/MnO_2_ batteries, the detrimental effect of Zn(OH)_4_^2−^ ion on the air-cathode has been reported to occur at a concentration of ≥ 0.1 M Zn(OH)_4_^2−^ ion [58]. Therefore, in addition to determination of this critical concentration, methods are required to reduce or completely avoid the crossover of Zn(OH)_4_^2−^ ions through the membrane. The strategy followed to minimize the crossover of Zn(OH)_4_^2−^ ions in this work is discussed in Section 3.2. Other membrane synthesis and modification strategies have been discussed in our recent review paper on the state-of-the-art membrane studies for Zn–air batteries [28].

### 2.2. Improving the Selectivity of Porous Membrane by Ion-Selective Polymers Coating

#### 2.2.1. Polymer and Cation Preparation

The preparation of PPO-Q polymer was carried out in two steps, the first step being benzylic bromination of a commercial PPO polymer. The successful bromination and quaternization of PPO polymer was confirmed by ^1^H NMR (Figure S1). DAPCl was also successfully synthesized starting from piperidine in a two-step process. The structure of prepared cationic monomer was confirmed by ^1^H NMR (Figure S2). In addition, FTIR spectroscopy has confirmed the successful synthesis of polymers and DAPCl cation (Figure S3).

#### 2.2.2. Modified Membrane Structural Characterization

**Figure 4:**
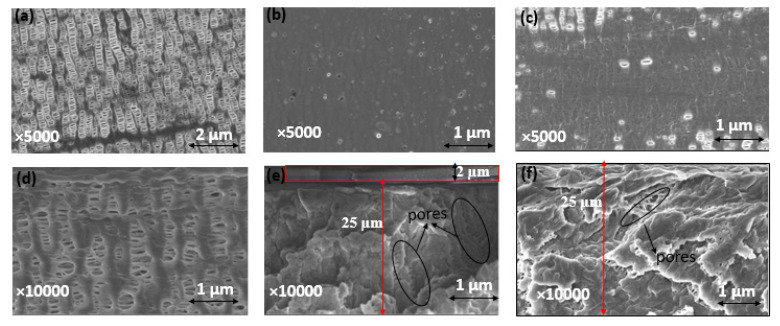
Surface SEM images of (**a**) Celgard^®^ 3501, (**b**) PPO-3.45 + 3501, (**c**) FAA + 3501 and SEM cross section view of (**d**) Celgard^®^ 3501, (**e**) PPO-3.45 + 3501, and (**f**) FAA + 3501 membranes. The densities of dense self-standing PPO-3.45 and FAA membranes were determined to be 1.17 and 1.14 g cm^−3^, respectively.

Celgard^®^ 3501 was chosen as a support membrane because of its low area resistance, high power density and commercial availability. Two solutions were cast on top of this support: (i) a mixture of PPO-Q and DAPCl with a theoretical IEC of 3.45 mmol OH^−^ g^−1^ (PPO-3.45) and (ii) a commercial FAA solution. SEM and FTIR analysis were used to confirm the successfulness of the PPO-3.45 coating. The top and cross-section of Celgard^®^ 3501 and modified membranes are shown comparatively in Figure 4. The pristine Celgard^®^ 3501 membrane displays porous structures (Figure 4a,d), in which the pores are lengthened and orientated in the same direction due to the dry unidirectional stretching of Celgard^®^ after the extrusion manufacturing process [59].

After the PPO-3.45 modification, the surface of Celgard^®^ 3501 membrane was homogeneously covered with the ionomer (Figure 4b). Considering the weight of PPO-Q and DAPCl used, an increase of 2.6 mg cm^−2^ was expected. However, the weight change recorded before and after drying and washing with water, the modified Celgard^®^ 3501 showed that only 2 (±0.1) mg cm^−2^ of ionomer was coated for the PPO-3.45 + 3501 membrane. The water-soluble part, analyzed by ^1^H NMR, consisted of unreacted DAPCl and poly(DAPCl) with a ratio of about 50:50. Considering that 0.6 mg cm^−^^2^ washed out with water, the IEC was recalculated to be 2.9 mmol OH^−^ g^−1^ ionomer, thus, 1.64 mmol OH^−^ g^−1^ of composite membrane.

The modified membrane showed an increase of about 2 μm in thickness compared to the pristine membrane, as measured by a micrometer. The same increase in thickness was observed in the SEM cross-section (Figure 4e). In addition, as shown in Figure 4e, it appears that part of the polymer is impregnated into the porous structure of the supporting membrane.

For the second modified membrane, a 0.8 g of FAA (12 wt. % in NMP) solution was cast on 64 cm^2^ of the porous membrane, thus, 1.5 mg cm^−2^ FAA polymer coating was expected. According to the manufacturer, the IEC of FAA-3–50 (membrane believed to be prepared from the FAA solution) is 1.6–2.1 mmol Cl^-^ g^−1^, which is equivalent to 1.65–2.2 mmol OH^−^ g^−1^. Approximately 80 (±2) wt.% of increase in the modified membrane was noted compared to the pristine one, corresponding to 1.2 mg cm^−2^ coating, thus, the IEC was recalculated to be 0.9–1.2 mmol OH^−^ g^−1^ of the composite membrane. However, no increase in thickness was noted by micrometer and SEM analysis, indicating the complete impregnation/penetration of the polymer solution onto the porous structure of the Celgard^®^ 3501 substrate [36]. Indeed, the SEM cross-section image shows that the pores are partially filled (Figure 4f).

Furthermore, Hg porosimetry measurements revealed that the total pore volumes of both PPO-3.45 + 3501 (0.64 cm^3^ g^−1^) and FAA + 3501 (0.72 cm^3^ g^−1^) membranes were lower than that of the pristine Celgard^®^ 3501 membrane (0.90 cm^3^ g^−1^). The drop of total porosity is seen as an indication of partial filling of the porous membrane with non-porous ionomers. Moreover, for both modified membranes, the larger pores seen in Celgard^®^ 3501, 100–200 nm range, seem to be filled by the ionomers as shown in Figure S4.

The coating and impregnation of the PPO-3.45 and total impregnation of FAA polymer solution could be due to the affinity/interaction between the ionomer and membrane surface in addition to the viscosity behavior of the polymer solution. Visually, the PPO-3.45 solution appears to be much more viscous than the FAA solution. To better understand the solution behavior during the casting, viscosity measurements of both PPO-DAPCl and FAA solutions are shown in Figure S5. PPO-DAPCl exhibits a non-Newtonian behavior. This means that viscosity depends on deformation applied to the material and in this case, PPO-DAPCl solution corresponds to a shear-thinning behavior in which viscosity decreases as the shear rate (deformation) applied to the sample increases. In the range of shear rates observed, viscosity drops from 7.0 to 0.14 Pa s, decreasing three orders of magnitude as shear rate increases. Nevertheless, it is worth noticing that for membrane casting using a doctor blade, shear rate corresponds to a magnitude of about 555 s^−1^ (calculated by dividing casting speed (1.7 cm s^−1^) by the 30 µm casting polymer solution used) corresponding to a high deformation regime. Using the data in Figure S5, viscosity is extrapolated to the representative shear rate and a value of 32 mPa is found. Contrastingly, the FAA solution shows a Newtonian behavior as viscosity remains constant with a value of 45.7 mPa s, which is comparable to the viscosity value of PPO-DAPCl in the range of shear rate investigated. At the casting shear rate, both solutions have comparable viscosities and, therefore, this can explain their similar behavior of large impregnation of the pore structure by the two ionomers.

#### 2.2.3. Electrolyte Uptake and Ion Conductivity

As can be seen from Table 4, after modifications, the electrolyte uptakes of both PPO-3.45 + 3501 and FAA + 3501 decreased significantly compared to the pristine Celgard^®^ 3501 membrane. Celgard^®^ 3501 was added in Table 4 for comparison purposes. The ion conductivities of PPO-3.45 + 3501 and FAA + 3501 membranes were found to be about 12 and 1 mS cm^−1^, respectively, at room temperature. The decline in conductivity of the modified membranes, compared to the pristine Celgard^®^ 3501, could be due to the filling of the pores, which greatly contributed to increase the resistance of these modified membranes, thus the ions (including OH^−^ ions) are less mobile to diffuse through the pores. The difference in conductivity between the two composite-membranes could be due to the difference in IEC between them. In addition to higher IEC, a self-standing membrane prepared from the PPO-3.45 has a much higher water uptake than the FAA membrane, so both ionic concentration and mobility are higher for PPO-3.45 + 3501 membrane compared to FAA + 3501.

#### 2.2.4. Alkaline Stability

Alkaline stability of the membranes is important in Zn–air batteries because the membranes have to work efficiently in the highly alkaline solution. The highly basic and nucleophilic OH^−^ may degrade the membrane. Therefore, stability of membranes in highly alkaline solution must be investigated prior to use in Zn slurry–air batteries. The stability of PPO-3.45 + 3501 membranes was investigated by comparing their structure before and after immersion in 6 M KOH aqueous solution for 10 days. FTIR analysis was used to investigate the alkaline stability of the membrane.

**Figure 5:**
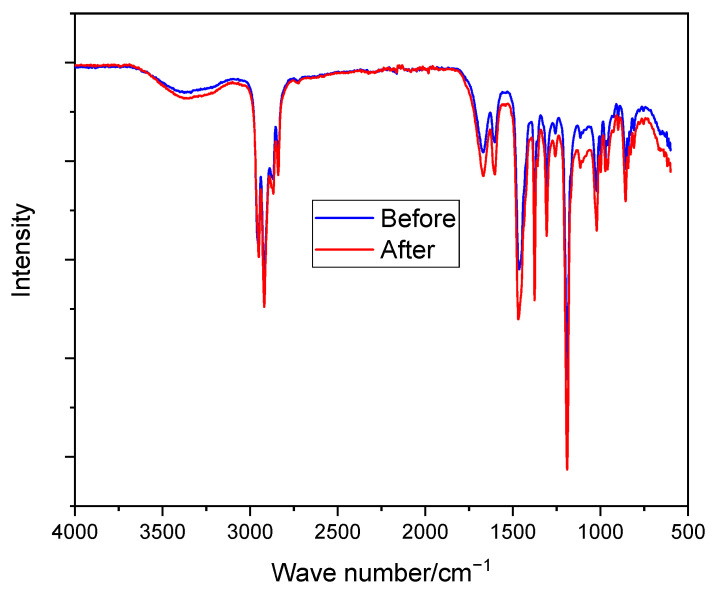
FTIR spectroscopy of PPO-3.45 + 3501 before and after immersion in 6 M KOH at room temperature for 10 days.

As shown in Figure 5, no new peaks of C–OH (above 3000 cm^−1^) were observed as a result of nucleophilic attack by the OH^−^ ion [39]. Moreover, the membranes do not show any significant changes in their spectrum (e.g., C–N shown at around 1300 cm^−1^) before and after immersion in the prepared solution, showing their stability in the alkaline medium. The good alkaline stability of *N*-spirocyclic cations employing AEMs can be explained by the high energy barrier value associated with the transition state during an OH^−^ attack [60,61].

#### 2.2.5. Zincate Ions Crossover

**Figure 6:**
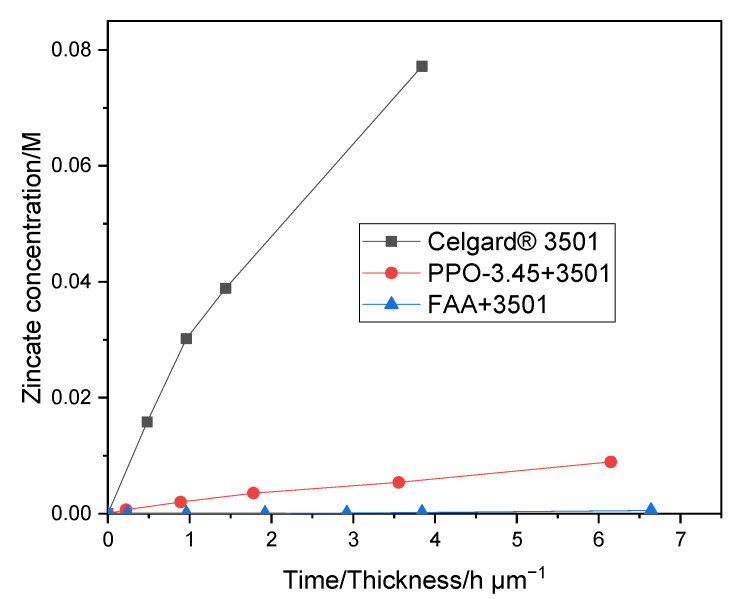
Zn(OH)_4_^2−^ crossover of Celgard^®^ 3501, and PPO-3.45 + 3501, FAA + 3501 as function of time/membrane thickness.

As shown in Figure 6, the PPO-3.45 + 3501 membrane exhibited a lower Zn(OH)_4_^2−^ crossover (0.015 M in 8 days) than that of Celgard^®^ 3501. The diffusion of Zn(OH)_4_^2−^ through the PPO 3.45 + 3501 membranes was significantly diminished due to filling of the pores and the thin coat layer on top of Celgard^®^ 3501 with ion selective polymer. With the addition of PPO-3.45 ionomer, the Zn(OH)_4_^2−^ ion diffusion coefficient decreased by a factor of 18. As shown in Figure 6, a much lower crossover of Zn(OH)_4_^2−^ ion through the FAA + 3501 membrane, compared to the PPO 3.45 + 3501 membranes, was observed which may be associated with the lower IEC and electrolyte uptake of FAA+ 3501 membrane. In the case of FAA modification, the Zn(OH)_4_^2−^ diffusion coefficient was reduced by a factor of more than 280. A large decrease of the Zn(OH)_4_^2−^ ions crossover was reached even if all the pores were not filled completely in the dry state, the properties of the ionomers in terms of water swelling and morphology have a significant impact on it. Two orders of magnitude reduction in Zn(OH)_4_^2−^ ions diffusion coefficient after coating Celgard^®^ 5550 membrane with polymerized ionic liquid has been reported elsewhere [37]. As a result, the lifetime of the battery was reported to increase by about 280% when compared to the pristine Celgard^®^ 5550-based battery (104 vs. 37 cycles).

Table 5 shows the Zn(OH)_4_^2−^ diffusion coefficient through the modified membranes. Zn(OH)_4_^2−^ ions diffusion coefficient through Celgard^®^ 3401 membrane modified with nanoparticles from the literature is included for comparison.

#### 2.2.6. Zn Slurry–Air Flow Battery Discharge Performance

The cell resistances employing the membranes in decreasing order are FAA+3501 (5.6 Ω cm^2^) > PPO-3.45 + 3501 (2.6 Ω cm^2^) > Celgard^®^ 3501 (2 Ω cm^2^). The significant increase in cell resistance of the FAA + 3501 membrane compared to the pristine Celgard^®^ 3501 could be due to the partial filling of the pores, which greatly contributed to the increase in resistance of the modified membrane, the OH^−^ ions are therefore less mobile to diffuse through the pores. Whereas, the PPO-3.45 + 3501 employing cell resistance slightly increased compared to the pristine membrane-based cell, probably due to the high IEC of the ionomer, allowing high OH^−^ ion conductivity and making it a good choice for the application.

**Figure 7:**
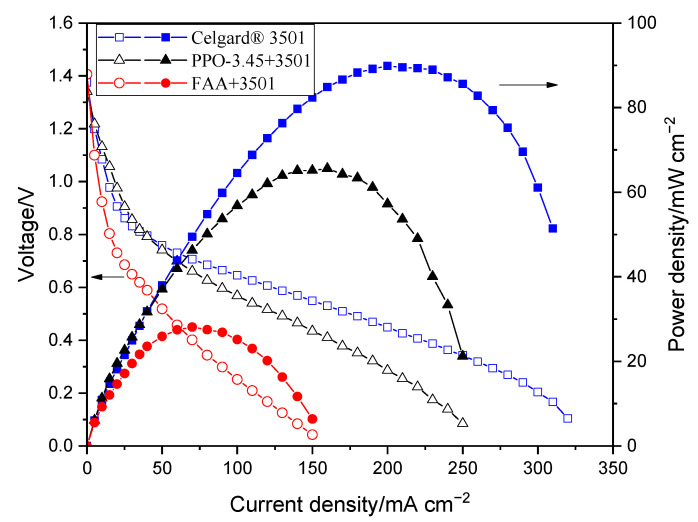
Polarization characteristics of the Zn slurry–air flow battery with the modified membranes.

The polarization characterizations of the modified membranes in Zn slurry–air battery are shown in Figure 7. The decrease in the power density of the impregnated membranes, compared to the pristine Celgard^®^ 3501 membrane, is due to the increase in resistance of the membranes associated with the filling of the pores with ionomers. The polarization characteristics revealed that PPO-3.45 + 3501-based cell produced a peak power density of 66 mW cm^−2^. Whereas, the lowest cell performance was obtained when the FAA + 3501 was used, which could be due to the low ion conductivity of the membrane. The reduction of internal resistance by a factor close to 3, in the case of FAA + 3501 membrane compared to the pristine membrane, had a high detrimental impact on the battery performance due to the ohmic drop associated with the high current density used.

## 3. Materials and Methods

### 3.1. Materials

PPO (Mn 20,000 and Polydispersity ~2.5) was purchased from Polysciences, Inc. Chlorobenzene (ACS reagent, ≥99.5%), *N*-methyl-2-pyrrolidone (NMP, reagent grade), tetrahydrofuran (THF, ACS, >99%), diethyl ether (>99%), 1,2-dichloroethane (99.8%), allyl bromide (98%), allyl chloride (98%) and chloroform (99.8%) were purchased from Alfa Aesar (Thermo Fisher, Kandel, Germany). Diallymethylamine (97%) and piperidine (≥99%) were bought from ABCR GmbH (Karlsruhe, Germany). Dimethyl sulfoxide-d_6_ (DMSO-d_6_, 99.9%) was supplied from Acros Organics ((Thermo Fisher, Kandel, Germany). *N*-bromosuccinimide (NBS, 99%), 2,2′-azobis(2-methylpropionitrile) (AIBN, 98%), methanol (99.9%) and chloroform-d (CDCl_3_-d, 99.9% D) were purchased from Sigma-Aldrich (Merck KGaA, Darmstadt, Germany). 2-hydroxy-4′-(2-hydroxyethoxy)-2-methylpropiophenone (Irgacure D-2959) was bought from Ciba Specialty Chemicals Inc (Basel, Switzerland). Cellophane™ 350PØØ was purchased from Futamura Chemical Co. Ltd. (Hamburg, Germany). Celgard^®^ membranes were kindly provided by Celgard, LLC (France). Zirfon^®^ and PBI^®^ were provided by AGFA Gevaert NV (Mortsel, Belgium) and Fumatech BWT GmbH (Bietigheim-Bissingen, Germany), respectively. All chemicals were used without further purification.

### 3.2. Polymer and Cation Preparation

PPO (6 g, 50 mmol PPO repeating unit) was dissolved in 60 mL of chlorobenzene in a 100 mL flask equipped with mechanical stirrer and a condenser under Ar gas. NBS (brominating agent) (2.07 g, 11.65 mmol) and AIBN (initiator) (0.115 g, 0.7 mmol) were added at 136 °C. Since PPO can be brominated on both its benzyl and aromatic positions, a benzyl position bromination was achieved by the high temperature used [62] while the extent of bromination was controlled by the amount NBS used [63]. The reaction continued at 136 °C for 3 h. The product was then precipitated in 600 mL of methanol drop wise. Finally, the product was filtered and dried at 60 °C in vacuum oven for 24 h. The obtained PPO-Br product (6.42 g, 96.8% yield) was confirmed by ^1^H NMR. The degree of bromination was determined by ^1^H NMR spectrum by comparing the integrals of the brominated methylene at 4.3 ppm and aromatic methyl group at 2.1 ppm. Subsequently, quaternized PPO polymer (PPO-Q) was prepared by reacting the PPO-Br polymer with diallylmethylamine [39]. PPO-Br (6.42 g) was dissolved in 150 mL THF in a 250 mL reaction flask. Diallylmethylamine was added in excess (300% molar in excess with respect to the Br units in the PPO-Br) to ensure full substitution of the Br units. The reaction continued for 48 h. The product was precipitated in diethyl ether drop wise, filtered and dried under vacuum at 35 °C overnight. The successful quaternization of the product was confirmed by ^1^H NMR.

The preparation of DAPCl was performed in two steps based on the method reported elsewhere with slight modifications [37].

### 3.3. Membrane Preparation

After the synthesis of the PPO-Q and DAPCl, coating over Celgard^®^ 3501 was performed as follows: 0.075 g PPO-Q, 0.0919 g DAPCl and 0.01023 g Irgacure D-2959 initiator were dissolved in 1,2-dichloroethane. Next, NMP (0.62 mL) was added and stirred for 30 min. The solution was covered with aluminum foil to avoid light induced initiator decomposition. The amount and ratio of DAPCl to PPO-Q (6:1, theoretical ion-exchange capacity (IEC) of 3.45 mmol OH^−^ g^−1^ polymer) was chosen based on preliminary optimization experiments to prepare a membrane with acceptable hydroxide ion conductivity. Once the dichloroethane was evaporated at room temperature, the remaining solution was poured on an 8 × 8 cm^2^ Celgard^®^ 3501 membrane and cast using doctor blade thickness of 30 µm. The coated membrane was degassed, crosslinked using UV irradiation for 3 min and dried overnight at 60 °C. The membrane prepared is denoted as PPO-3.45 + 3501.

In a separate fabrication experiment, 0.8 g of Fumion FAA-3-SOLUT-10 (12 wt.%, determined by drying at 80 °C for 24 h in this study) (referred to as FAA) was coated on an 8 × 8 cm^2^ Celgard^®^ 3501 using doctor blade thickness of 30 µm. Similarly, the FAA modified membrane (FAA + 3501) was dried overnight at 60 °C.

### 3.4. Characterization

#### 3.4.1. Structural Characterization

Chemical structures and purity of the polymer and monomer were determined by ^1^H NMR spectroscopy using CDCl_3_, deuterium or DMSO-*d*_6_ as solvents in the Bruker Ascend^TM^ 400 MHz Spectrometer. The polymer coating was confirmed by Bruker’s VERTEX 70v FT-IR Spectrometer in range of 4000–500 cm^−1^ with resolution of 2 cm^−1^. SEM analysis was done to study the homogeneity of the coating.

#### 3.4.2. Electrolyte Uptake

Electrolyte uptake of membranes was determined by immersing the membranes in 6 M aqueous KOH for 24 h at room temperature. Membrane samples were taken out from the solution and their surface solution was removed to record their wet weight. The liquid electrolyte uptakes of the membranes were calculated from the difference of wet and dry weights of the membrane samples based on the following equation (Equation (1)):

(1) $$\mathrm{KOH}\;\mathrm{uptake}\left(\%\right)=\frac{W_{\mathrm{wet}}-W_{\mathrm{dry}}}{W_{\mathrm{dry}}}\times 100$$

where W_dry_ and W_wet_ are the weights of the membranes before and after absorbing the liquid electrolyte.

Volume swelling degree: dried membranes were immersed in 6 M KOH for 24 h at room temperature and the volume (area x thickness) of all the membranes was measured. The difference between the wet volume and dry volume was used to calculate the swelling ratio of the membranes according to the following Equation (2):

(2) $$\Delta V\;\left(\%\right)=\frac{\left(V_{\mathrm{wet}}-V_{\mathrm{dry}}\right)}{V_{\mathrm{dry}}}\times 100$$

#### 3.4.3. Ionic Conductivity

The ionic conductivity of the membranes was measured by electrochemical impedance spectroscopy (EIS) in the frequency range 13 MHz–5 Hz at room temperature. All membranes were immersed in 6 M KOH solutions for 24 h before measuring their conductivity. The membranes were taken out from the electrolyte solution, the surface KOH aqueous solution was removed and measured for their resistance. The membrane ionic conductivity (σ) was calculated by the following formula (Equation (3)):

(3) $$\sigma =\frac{l}{\mathrm{RA}}$$

where l is the thickness of the membrane (cm), A is the active area of the membrane sandwiched between two electrodes (0.0314 cm^2^) and R is the electric resistance of the membrane (Ω).

#### 3.4.4. Rheometry

Rotational rheometry was performed using a stress-controlled rheometer Discovery HR-3 (TA instruments). PPO-DAPCl viscosity was determined with a cone-plane geometry (D = 60 mm, 1° angle), whereas FAA viscosity was determined using a bob-in-cup geometry (bob diameter = 28 mm, bob length = 42 mm and cup diameter = 30 mm), due to sample lower viscosity. Measurements were performed at 25 °C with the aid of a Peltier plate integrated system and an anti-evaporation tool was used to prevent changes in sample properties. Steady state measurements were obtained by applying a constant shear rate from 0.01 to 100 s^−1^ and when shear stress (or torque) signal was stabilized, the measurement was taken. Validation of measurement was performed by applying decreasing steps from 100 to 0.01 s^−1^ and no significant differences were found. Lower shear rates resulted in torque values that were outside the rheometer’s range of measurement, so these values were not included.

#### 3.4.5. Membrane Density

The density (ρ) of the membranes was measured by density measurement kit (Mettler-Toledo), which contains weighting pans at ambient and immersed in a solvent at 20 °C using toluene as liquid phase. The prepared membranes in OH^-^ form were dried at 60 °C under vacuum for 24 h.

The membranes density in g cm^−3^ was calculated by the following Equation (4):

(4) $$\rho =\frac{m_{\mathrm{ambient}}}{m_{\mathrm{ambient}}-m_{\mathrm{toluene}}}\times \rho_{\mathrm{toluene}}$$

where m_ambient_ and m_toluene_ are weights of the membrane at ambient and in toluene, respectively.

ρ_toluene_ is density of toluene (0.87 g mL^−1^).

#### 3.4.6. Mercury Porosimetry

Mercury (Hg) intrusion measurements (Quantachrome PoreMaster) were used to determine the intruded volume, volume of pores/mass of membrane (cm^3^ g^−1^) of the pristine and modified membranes.

#### 3.4.7. Alkaline Stability

The alkaline stability of the modified membrane (PPO-3.45 + 3501) was studied by immersing the membranes in a typical solution used in Zn–air batteries i.e., 6 M KOH for 10 days at room temperature. The electrolyte was replaced every 48 h. The stability of the membranes was investigated by comparing the structure (by FTIR analysis) of the membranes before and after immersion in the alkaline solution.

#### 3.4.8. Zincate Ion Crossover

The crossover of Zn(OH)_4_^2−^ ions through both the commercial and modified membranes was tested using a self-made diffusion cell (Figure S6). The enriched side of the diffusion cell consists of 0.3 M of Zn(OH)_2_ dissolved in 6 M aqueous KOH solution (15 mL). Whereas, the deficiency chamber was filled with only 6 M KOH aqueous solution (15 mL). The membrane sample was placed between the two compartments. At predefined period of times, a 0.1 mL sample was taken from the right-side chamber. The time-dependent concentration of Zn(OH)_4_^2−^ ions in the right chamber was then determined using atomic absorption spectroscopy (AAS, PinAAcle^™^ 900F, PerkinElmer (Waltham, MA, U.S)). A wavelength of 213.86 nm and 0.7 nm slit was used to determine the concentration of Zn in each sample solution. The diffusion coefficients (D) of Zn(OH)_4_^2−^ ions of the different membranes were calculated from the following Equation (5) [64]:

(5) $$V_{B}\frac{{\mathrm{dC}}_{B}\left(t\right)}{\mathrm{dt}}=\frac{\mathrm{DA}}{L}(C_{A}-C_{B}\left(t\right)$$

After integration, by assuming volume of the right chamber (*V_B_*) does not change with time, Equation (5) can be changed to:

(6) $$\ln\left(\frac{C_{A}}{C_{A}-C_{B}}\right)=\frac{\mathrm{DA}}{V_{B}L}t$$

where D is the diffusion coefficient of Zn(OH)_4_^2−^ ions through the membrane (m^2^ min^−1^), t is the time (min), A is the effective area of the membrane (~0.5 cm^2^), L is the thickness of the membrane (m), C_A_ and C_B_ are the zincate concentrations (mol L^−1^) in the enriched and deficiency chambers, respectively.

#### 3.4.9. Single Cell Assembly and Electrochemical Performance

**Figure 8:**
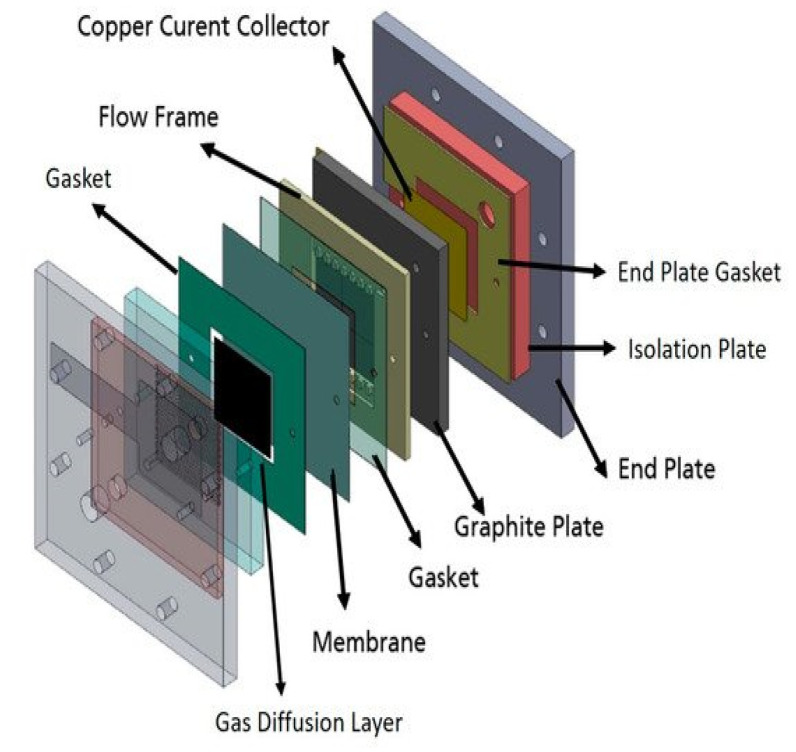
Schematic representation of the single cell Zn slurry–air flow battery with a flow frame [66].

A single cell used for this study is identical to the best performing cell design reported in a previous study [43], which has a serpentine flow field of CuNi plate (Figure 8). A catalyst coated electrode (CCE) was used for the air cathode. The catalyst ink was prepared by mixing a Pt/C catalyst (40% Pt, Alfa Aesar, (Thermo Fisher, Kandel, Germany)) with Pt loading at 1 mg cm^−2^, isopropanol and deionized water. The prepared ink was then sonicated in an ultrasonic water bath for 15 min and sprayed onto a geometric area of 25 cm^2^ (5 × 5 cm and 0.0235 cm thickness) gas diffusion layer (SGL Carbon, 29BC, FuelCellStore, College Station, TX, USA). The CCE was placed between the membranes and the cathode bipolar plate. The Zn slurry (Table 6) was prepared using the same method and chemicals as in a previous study [65]. Each solution was mixed at 4000 rpm for 3 min.

The diameters of zinc particles were measured using a laser diffraction analyzer (Mastersizer 2000, Malvern Panalytical (Malvern, United Kingdom). The zinc used in this study can be classified as microparticles, with a mass-median-diameter D_50_ = 65.5 µm. Only 10% of the particles are smaller than 46.5 µm.

In order to determine electrochemical performances of each membrane, current–voltage characteristic curves (polarization curves) and EIS were measured. BaSyTec GSM Battery Test System (BaSyTec GmbH, Asselfingen, Germany) was used for current–voltage characterization while Zahner IM6 workstation was used for impedance spectra. The cell resistance of the battery employing the membranes was determined from the slope of the current density–voltage (IV) curves. In addition, the ohmic resistances of the cell were assessed by EIS measurements which were carried out at 1.3 V. All membranes were immersed in 1 M KOH solutions for 24 h before measuring their resistance. During the measurements, Zn slurry was flowing into anode compartment at 160 mL min^−1^ flow rate while synthetic air was flowing into cathode at 100 mL min^−1^ flow rate. At each current density, the voltage was recorded for 30 s and averaged due to the fluctuation of a slurry electrode flowing into the single cell. Figure 8 presents the schematic representation of the Zn slurry–air flow battery used.

## 4. Conclusions

The order of peak power density of the cell employing the commercial membranes in decreasing order was Celgard^®^ 3501 > Cellophane™ 350 PØØ ≈ Celgard^®^ 3401> Celgard^®^ 5550 > Zirfon^®^ > PBI^®^, in agreement with their respective cell resistances. However, the membranes with good polarization characteristics of Zn slurry–air battery, the Cellophane™ 350 PØØ and the three Celgard^®^ membranes showed a significant crossover of the soluble Zn(OH)_4_^2−^ ions (diffusion coefficient being greater than 6.5 × 10^−12^ m^2^ s^−1^). To reduce the crossover of Zn(OH)_4_^2−^ ions through the porous membranes, Celgard^®^ 3501 was modified using two different ion-selective polymers (Supplementary Materials Table S1). In PPO-3.45 + 3501, the polymers were found to be coated and impregnated on the support membrane. Whereas, in the second work, the FAA polymer impregnated into the porous structure of the commercial membrane. Compared to the pristine Celgard^®^ 3501, the PPO 3.45+ 3501 membrane showed 18-fold lower crossover of Zn(OH)_4_^2−^ ions (5.2 × 10^−13^ vs. 9.2 × 10^−12^ m^2^ s^−1^). The modified membrane-based battery delivered a high maximum power density 66 mW cm^−2^, lower than that of Celgard^®^ 3501-based cell (90 mW cm^−2^) due to the increase in resistance of the membrane associated with the partial filling of the pores with ionomers. In summary, modified membranes are promising candidates for use in rechargeable Zn slurry–air flow batteries, thus further optimizations are required.

## Figures and Tables

**Table 1:** Properties of the commercial membranes tested in this work.

Membrane	Material	Structure	Pore Size (nm)	Porosity (%)	Thickness (µm)	Ref.
Celgard^®^ 3501	PP	Monolayer and surfactant-coated	64	55	25	[40]
Celgard^®^ 3401	PP	Monolayer and surfactant-coated	43	41	25	
Celgard^®^ 5550	PP	Laminated and surfactant-coated	64	55	70	
Cellophane™ 350 PØØ	Cellulose	Negatively charged	-	-	86	
PBI^®^	Polybenzimidazole		-	-	8	
Zirfon^®^	Polysulfone and ZrO_2_	Porous composite diaphragm	150 ± 50	55 ± 10	500 ± 50	[41,42]

**Table 2:** Characterization of commercial membranes: electrolyte, swelling degree and conductivity.

Membrane	Electrolyte Uptake (wt. %)	Percent (%) of Porosity Filled with Electrolyte *	Swelling Degree: ΔV (%)
Celgard^®^ 3501	98 ± 2	76	3
Celgard^®^ 3401	49 ± 2	63	3.6
Celgard^®^ 5550	113 ± 3	82	4.1
Cellophane™ 350 PØØ	129 ± 3	**	3.2
PBI^®^	36 ± 0.4	**	1.2
Zirfon^®^	51 ± 0.5	89	3.1

* Percent of $\mathrm{porosity}\;\mathrm{filled}\;\mathrm{with}\;\mathrm{electrolyte}\;=\;\frac{\mathrm{Porosity}\;\mathrm{filled}\;\mathrm{with}\;\mathrm{electrolyte}}{\mathrm{Volume}\;\mathrm{porosity}}\;=\;\frac{\frac{\left(\frac{\mathrm{electrolyte}\;\mathrm{uptake}\;\left(\%\right)}{1.26\frac{g}{\mathrm{cm}3}}\right)}{\left(\frac{\mathrm{electrolyte}\;\mathrm{uptake}\;\left(\%\right)}{1.26\frac{g}{\mathrm{cm}3}}\right)\;+\;\left(\frac{100}{\mathrm{polymer}\;\mathrm{density}}\right)}}{\mathrm{volume}\;\mathrm{porosity}\;\left(v/v\right)}\;*\;100$ (Density used: PP = 0.92 g cm^−^³ and Zirfon^®^ = 2.37 g cm^−^³, apparent density [47]) and volume porosity are provided in Table 2. ** Porosity filled with the electrolyte are not determined as their porosity volumes are not available.

**Table 3:** Diffusion coefficients of Zn(OH)_4_^2−^ ions through the membranes.

Membrane	D Zn(OH)_4_^2−^ (m^2^ s^−1^)	Ref
Celgard^®^ 3501	9.2 × 10^−12^	This work
Celgard^®^ 3401	6.6 × 10^−12^	
Celgard^®^ 5550	1.4 × 10^−11^	
Cellophane™ 350 PØØ	1.3 × 10^−11^	
Zirfon^®^	6.6 × 10^−11^	
PBI^®^	ND *	
Celgard^®^ 3501	3.2 × 10^−11^	[31]
	1.3 × 10^−11^	[52]
	9.5 × 10^−12^	[53]
Cellophane™ 350 PØØ	3.8 × 10^−12^	[54]
	6.7 × 10^−12^	[52]
	3.3 × 10^−12^	[53]

* Not detected, too low to be detected by AAS within a week of experiment.

**Table 4:** Characterization of the prepared membranes: electrolyte and ion conductivity.

Membrane	Electrolyte Uptake (wt.%)	Ion Conductivity (mS cm^−1^)
Celgard^®^ 3501	98 ± 2	17 ± 2.5
PPO-3.45 +3501	55 ± 1.9	12 ± 0.9
FAA + 3501	46 ± 2.1	1 ± 0.7

**Table 5:** Zincate ions diffusion coefficient through the modified membranes.

Membrane	Diffusion Coefficient (m^2^ s^−1^)	Ref.
Celgard^®^ 3501	9.2 × 10^−12^	This work
PPO-3.45 + 3501	5.2 × 10^−13^	
FAA + 3501	3.3 × 10^−14^	
Two Celgard^®^ 3401 coated with Mn(OH)_2_	6.0 × 10^−15^	[34]

Two Celgard^®^ 3401 membranes used together had a diffusion coefficient of Zn(OH)_4_^2−^ of 6.9 × 10^−12^ m^2^ s^−1^ [35].

**Table 6:** Composition of the Zn slurry used.

Chemicals	Mass Fraction (wt. %)
Zn	33.8
ZnO	4
Carbopol	0.7
KOH + H_2_O	61.5

## Data Availability

The raw data and samples presented in this study are available on request from the corresponding authors. All relevant processed data is shown in the manuscript and Supplementary Materials associated.

## Supplementary Materials

The following are available online, Figure S1: ^1^H NMR spectra of PPO-Br (m = 0.15) (a) in CDCl_3_ and PPO-Q (b) in DMSO-d_6_, Figure S2: ^1^H NMR spectra of N-allylpiperidine in CDCl_3_ (i) and ^1^H NMR (ii) of spectra DAPCl in d-H_2_O, Figure S3: FTIR spectra of (a) PPO, PPO-Br, PPO-Q and (b) DAPCl [66,67,68,69]. Figure S4: Measurements of Hg intrusion porosity for Celgard^®^ 3501 and modified membranes. Normalized pore volume as a function of pore diameter of Celgard^®^ 3501, FAA + 3501 and PPO-3.45 + 3501. Data show closure of larger Celgard^®^ 3501 pores as a result of impregnation with the ionomers. Figure S5: Viscosity as function of shear rate for polymer solutions at 25 °C. Figure S6. Image of the diffusion cell used. Table S1: Summary of diffusion coefficient through the membranes and their cell performance (resistance, peak power density) [66,67,68,69].

## Sample Availability

Not available.
