# Supplementary material for: Pristine and Modified Porous Membranes for Zinc Slurry–Air Flow Battery
Source: Molecules. 2021 Jul 2;26(13):4062. doi: 10.3390/molecules26134062 (PMC8272061; doi:10.3390/molecules26134062)
Supplement: Supplementary file 1 [file molecules-26-04062-s001.zip › molecules-1235318-supplementary.pdf]

Supplementary Material

Pristine and Modified Porous Membranes for Zinc Slurry–Air Flow Battery

Misgina Tilahun Tsehaye [1], Getachew Teklay Gebreslassie [1], Nak Heon Choi [2,3], Diego Milian [4], Vincent Martin [1], Peter Fischer [2], Jens Tübke [2,3], Nadia El Kissi [4], Mateusz L. Donten [5], Fannie Alloin [1,6,*] and Cristina Iojoiu [1,6,*]

1 Univ. Grenoble Alpes, Univ Savoie Mont Blanc, CNRS, Grenoble INP, LEPMI, 38000 Grenoble, France; misgina-tilahun.tsehaye@grenoble-inp.fr (M.T.T.); getachewtek0@gmail.com (G.T.G.); vincent.martin@lepmi.grenoble-inp.fr (V.M.)

2 Applied Electrochemistry, Fraunhofer Institute for Chemical Technology ICT, Joseph-von-Fraunhofer, Straße 7, 76327 Pfinztal, Germany; nak.choi@ict.fraunhofer.de (N.H.C.); peter.fischer@ict.fraunhofer.de (P.F.); jens.tuebke@ict.fraunhofer.de (J.T.)

3 Institute for Mechanical Process Engineering and Mechanics, Karlsruhe Institute of Technology KIT, Straße am Forum 8, 76131 Karlsruhe, Germany

4 Univ. Grenoble Alpes, CNRS, Grenoble INP, LRP, 38000 Grenoble, France; diego.milian@univ-grenoble-alpes.fr (D.M.); Nadia.ElKissi@ujf-grenoble.fr (N.E.K.)

5 Amer-Sil S.A., 61 Rue d’Olm, 8281 Kehlen, Luxembourg; mateusz.donten@amer-sil.com

6 Réseau sur le Stockage Electrochimique de l’Energie (RS2E), CNRS, FR3459, CEDEX 80039 Amiens, France

* Correspondence: Fannie.Alloin@grenoble-inp.fr (F.A.) and Cristina.iojoiu@lepmi.grenoble-inp.fr (C.I.)

Polymer and cation synthesis

NMR:

PPO bromination was performed using AIBN and NBS as initiator and brominating agent, respectively at 136 °C. The successful bromination of PPO polymer (PPO-Br) was confirmed by ^{1}H NMR. The PPO-Br (Figure S1a) shows signals at 4.3 ppm which corresponds to the CH_2Br units in the benzylic position. The signals between 6.5 and 6.7 ppm corresponds to the aromatic protons. The degree of bromination was calculated based on the ^{1}H NMR spectrum by comparing the ratio of the integrated signals of the CH_2Br protons at 4.3 ppm and the protons in aromatic CH_3 at 1.8 ppm. Figure S1b shows the ^{1}H NMR signals of PPO-Q where not only the signals for the proton from CH_2Br at 4.3 ppm are disappeared but also new chemical shifts (6.3-5.4 and 4.5-3.75 ppm) are appeared attributing the newly integrated protons from the ammonium groups. The integration of these new signals confirms the complete substitution of Br in CH_2Br with methyldiallyl ammonium bromide.

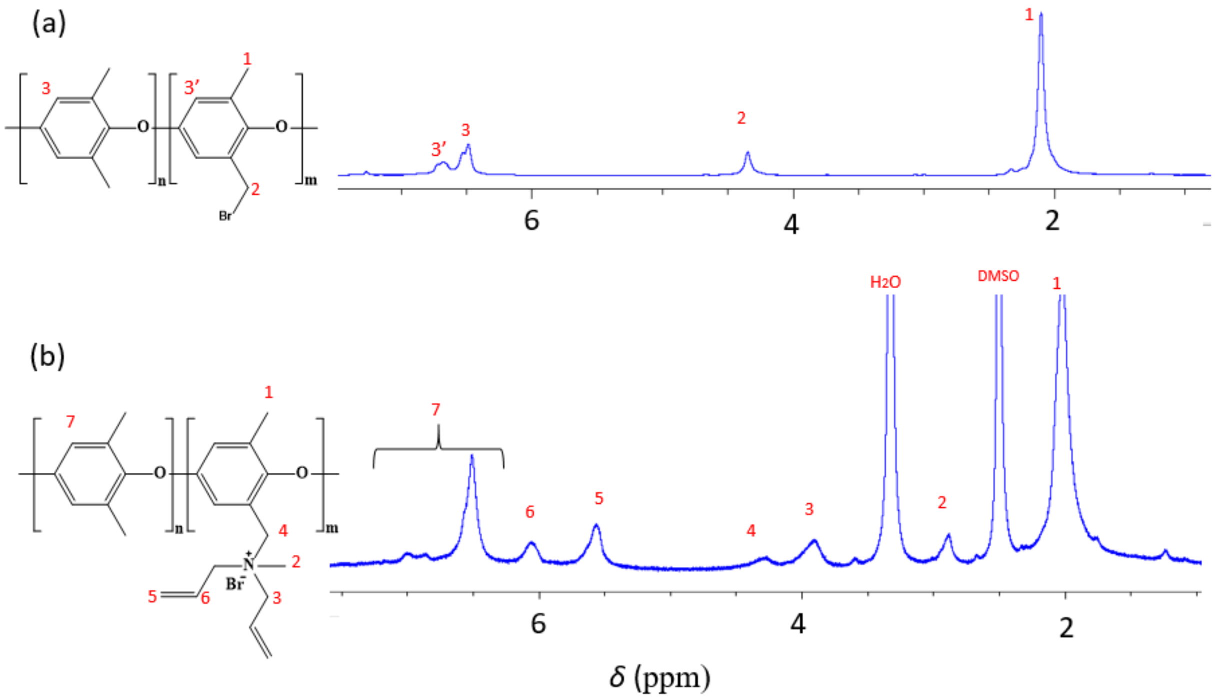

Figure S1. ^{1}H NMR spectra of PPO-Br (m = 0.15) (**a**) in $CDCl_3$ and PPO-Q (**b**) in DMSO-d_6.

The structure of prepared cationic monomer was confirmed by ^{1}H NMR. Figure S3b shows the NMR spectrum of DAPCl, which shows the doubling of integral of protons in 5.9-6.1 ppm and a shift of the peaks for the protons at 3.9 ppm and 3.4 ppm which all belong to the protons in DAPCl.

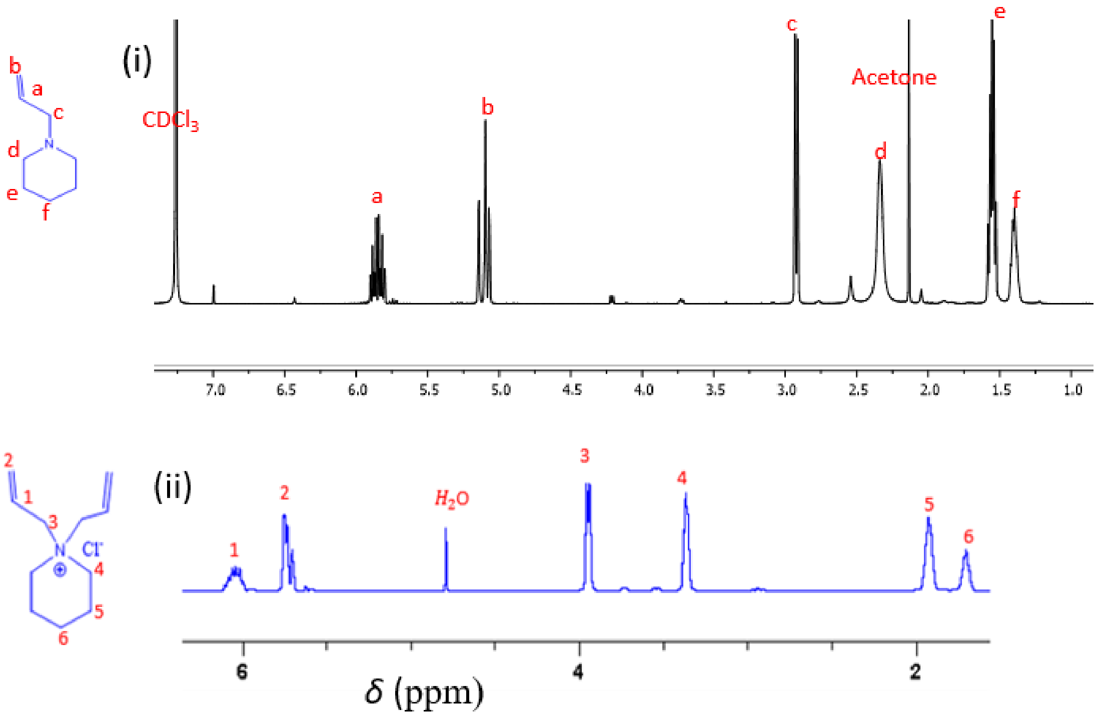

Figure S2. ^{1}H NMR spectra of *N*-allylpiperidine in $CDCl_3$ (**i**) and ^{1}H NMR (**ii**) of spectra DAPCl in d-H_2O.

FTIR

Characteristic peaks of C=C stretching and C-H stretching of benzene ring in PPO appeared at around 1600 cm^{-1} and 1470 cm^{-1}, respectively [66,67]. A new characteristic peak at 987 cm^{-1}, which is attributed to the C-Br groups [68] was observed in the FTIR spectra of PPO-Br. After quaternization, the C-Br peak disappeared which confirms the reaction of CH_2Br side chains of PPO with diallylmethylamine to form the PPO-Q. The characteristic peaks in DAPCl, including C-N stretching, and quaternary ammonium are shown around 1300 cm^{-1} and 955 cm^{-1}, respectively [69]. Moreover, the C-H stretching, C=C stretching, and CH_2 bending are shown at 2977 cm^{-1}, 1635 cm^{-1}, and 1456 cm^{-1} respectively [69].

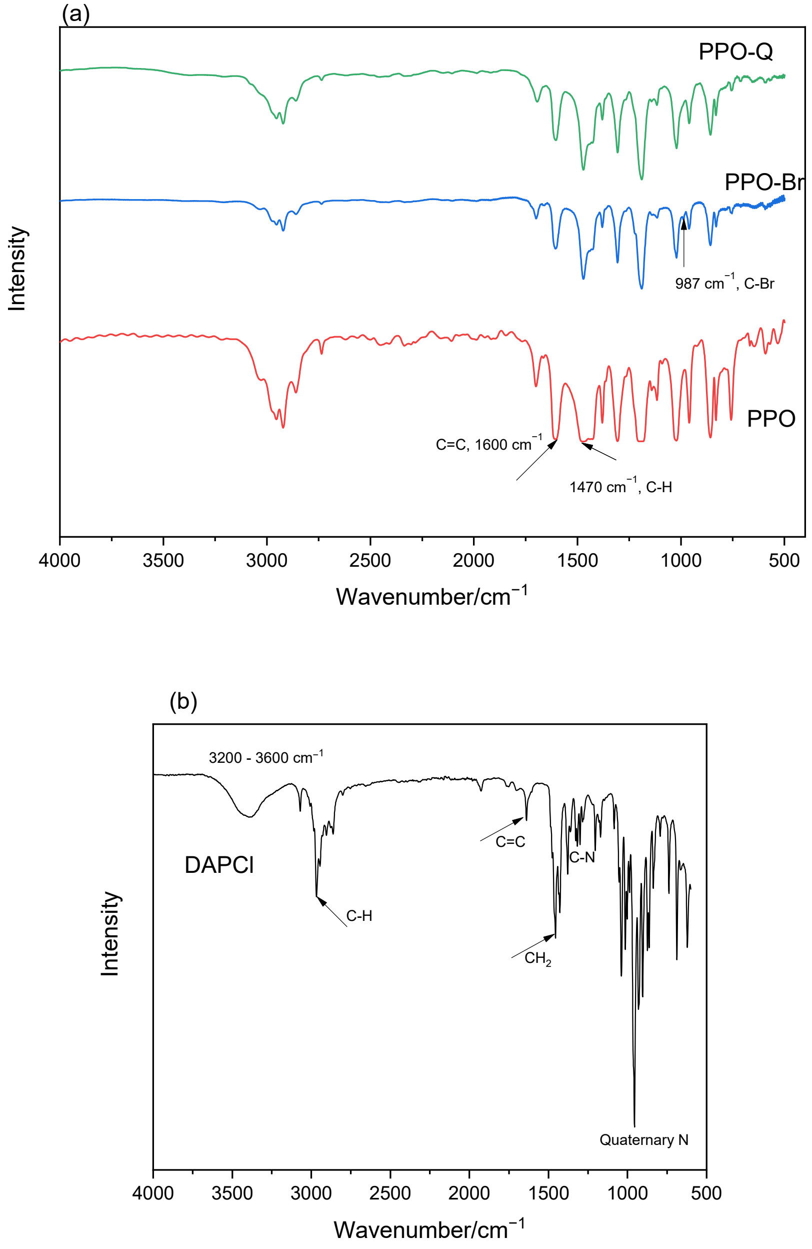

Figure S3. FTIR spectra of (**a**) PPO, PPO-Br, PPO-Q and (**b**) DAPCl. The spectral bands from 3600 – 3200 cm^{-1} are characteristic peaks of OH^- groups absorbed in the different samples.

Pore filled with ionomers: Hg porosimetry

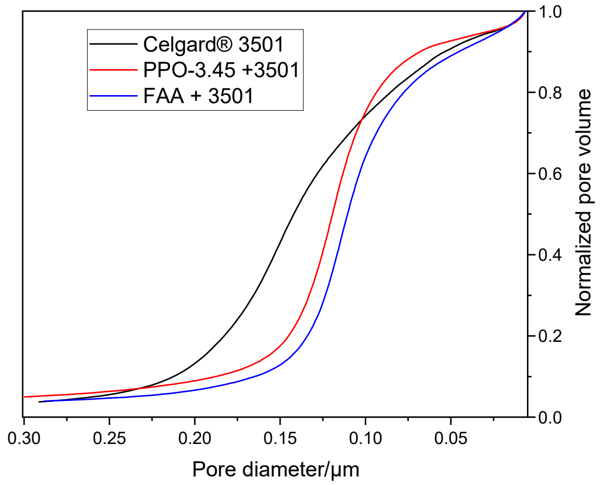

Figure S4. Measurements of Hg intrusion porosity for Celgard® 3501 and modified membranes. Normalized pore volume as a function of pore diameter of Celgard® 3501, FAA + 3501 and PPO-3.45 + 3501. Data show closure of larger Celgard® 3501 pores as a result of impregnation with the ionomers.

Rheology

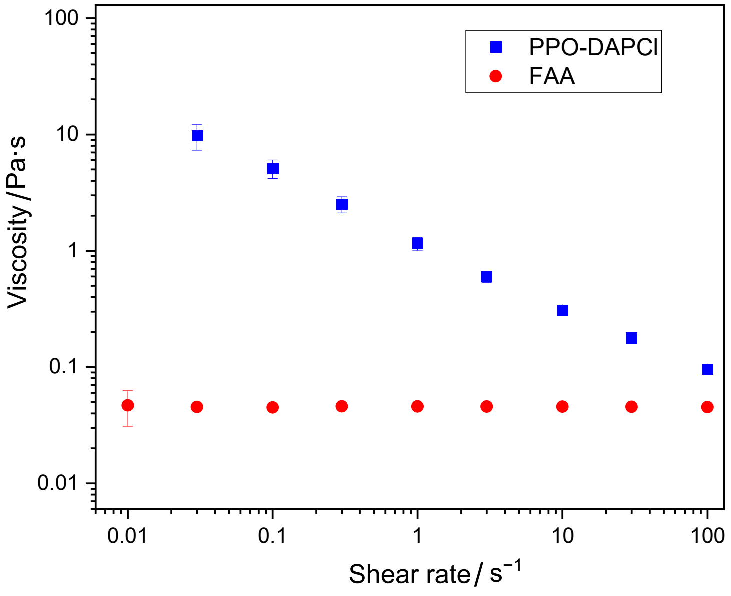

Figure S5. Viscosity as function of shear rate for polymer solutions at 25 °C.

Diffusion cell used

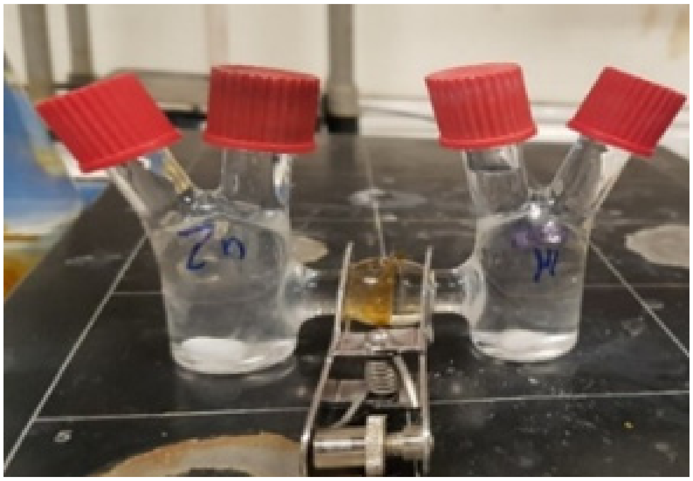

Figure S6. Image of the diffusion cell used.

Table S1. Summary of diffusion coefficient through the membranes and their cell performance (resistance, peak power density).

Membranes	D $Zn(OH)_4^{2-}$ (m^2 s^{-1})	Cell resistance (Ω cm^2)	Peak power density (mW cm^{-2})
Celgard® 3501	9.2×10^{-12}	2	90
Celgard® 3401	6.6×10^{-12}	2.4	69
Celgard® 5550	1.4×10^{-11}	3.2	58
Cellophane™ 350 PØØ	1.3×10^{-11}	2.5	72
Zirfon®	6.6×10^{-11}	3.9	44
PBI®	ND*	5.5	32
PPO-3.45 +3501	5.2×10^{-13}	2.6	66
FAA + 3501	3.3×10^{-14}	5.6	28

*ND: Not determined

References

66. Msomi, P.F.; Nonjola, P.; Ndungu, P.G.; Ramonjta, J. Quaternized poly(2.6 dimethyl-1.4 phenylene oxide)/polysulfone blend composite membrane doped with ZnO-nanoparticles for alkaline fuel cells. *J. Appl. Polym. Sci.* **2018**, *135*, 45959, doi:10.1002/app.45959.

67. Yang, Y.; Xu, Y.; Ye, N.; Zhang, D.; Yang, J.; He, R. Alkali Resistant Anion Exchange Membranes Based on Saturated Heterocyclic Quaternary Ammonium Cations Functionalized Poly(2,6-dimethyl-1,4-phenylene oxide)s. *J. Electrochem. Soc.* **2018**, *165*, F350–F356, doi:10.1149/2.1031805jes.

68. Zhu, L.; Zimudzi, T.J.; Wang, Y.; Yu, X.; Pan, J.; Han, J.; Kushner, D.I.; Zhuang, L.; Hickner, M.A. Mechanically Robust Anion Exchange Membranes via Long Hydrophilic Cross-Linkers. *Macromolecules.* **2017**, *50*, 2329–2337, doi:10.1021/acs.macromol.6b01381.

69. Patel, A.M.; Patel, R.G.; Patel, M.P. Nickel and copper removal study from aqueous solution using new cationic poly[acrylamide/ N,N -DAMB/ N,N -DAPB] super absorbent hydrogel. *J. Appl. Polym. Sci.* **2011**, *119*, 2485–2493, doi:10.1002/app.32818.
